# Clinical Observations of Psychiatric and Sexual Outcomes in Patients with Trazodone-Associated Ischemic Priapism

**DOI:** 10.3390/medicina62040612

**Published:** 2026-03-24

**Authors:** Hubert Dąbrowski, Tomasz Ząbkowski, Kamil Ciechan, Marcin Wajszczuk, Hubert Andrzej Krzepkowski, Tomasz W. Kaminski, Patryk Uciechowski, Tomasz Syryło

**Affiliations:** 1 Świętokrzyskie Center of Psychiatry in Morawica, Psychiatric Ward of the Mental Health Center in Kielce, 59 Kosucińskiego Street, 25-450 Kielce, Poland; 2 Department of General, Functional and Oncological Urology, Military Institute of Medicine–National Research Institute, 128 Szaserów Street, 04-141 Warsaw, Polandhkrzepkowski@wim.mil.pl (H.A.K.); puciechowski@wim.mil.pl (P.U.);; 3 Warsaw Bar Association, 15/16 Żytnia Street, 01-014 Warsaw, Poland; 4 Hemostasis and Thrombosis Program, Versiti Blood Research Institute, Milwaukee, WI 53226, USA

**Keywords:** trazodone, ischemic priapism, psychiatric outcomes, sexual dysfunction, adverse drug reaction

## Abstract

*Background and objectives*: Ischemic priapism is a rare but serious adverse effect of trazodone, associated with a high risk of long-term sexual dysfunction. While its urological consequences are well described, psychiatric and psychosocial outcomes remain insufficiently explored. This study assessed psychiatric and sexual sequelae following trazodone-associated ischemic priapism and compared clinical characteristics with trazodone-treated patients without priapism. *Materials and Methods*: In this single-center observational study, 268 adult patients receiving trazodone were analyzed, including 17 patients with ischemic priapism and 251 controls. Data on episode duration and urological management were collected. Psychiatric status and sexual functioning were evaluated through structured clinician-led interviews informed by validated psychometric frameworks during hospitalization and at 1-, 3-, and 6-month follow-up. Nonparametric analyses and Spearman rank correlations were applied. *Results*: Patients with priapism were significantly older than controls (44.1 ± 5.1 vs. 39.0 ± 4.4 years; *p* < 0.0001), while trazodone dose distribution did not differ between groups. The mean episode duration was 26.5 ± 16 h (median 24 h). Older age and longer ischemic duration were independently associated with increased treatment intensity, whereas trazodone dose was not. Persistent depressive and anxiety symptoms and impaired sexual functioning were observed in a subset of patients during follow-up. *Conclusions*: Trazodone-associated ischemic priapism is not only an acute urological emergency but may also lead to sustained psychiatric and sexual sequelae. Interdisciplinary follow-up should be considered to address long-term psychosocial outcomes.

## 1. Introduction

Trazodone is an antidepressant classified as a serotonin antagonist and reuptake inhibitor (SARI). Although it was originally developed for the treatment of major depressive disorder, its role in contemporary clinical practice has expanded substantially. At present, trazodone is frequently prescribed for sleep-related disorders, anxiety symptoms, and affective complaints accompanying somatic conditions, particularly among older adults. The popularity of trazodone stems from its relatively favorable safety profile, low potential for dependence, and limited anticholinergic activity compared with other psychotropic medications [1].

The adverse effect profile of trazodone is most often characterized by central nervous system sedation, episodes of dizziness, postural blood pressure drops, and gastrointestinal discomfort. More rarely, disturbances in cardiac conduction, transient loss of consciousness, and prolonged penile erection have been reported. Although the occurrence of priapism is uncommon, it remains among the most consistently described serious adverse reactions associated with trazodone and constitutes a clinically relevant issue given the potential for lasting physical and psychological sequelae [2].

The mechanism of trazodone-induced priapism is primarily attributed to antagonism of α1-adrenergic receptors, resulting in impaired detumescence and sustained relaxation of the smooth muscle of the corpora cavernosa. An additional contributory role is attributed to modulation of serotonergic neurotransmission, which affects both central and peripheral regulation of sexual function. In contrast to other antidepressants, trazodone is cited disproportionately often in reports of drug-induced priapism [3].

Priapism refers to a persistent penile erection that continues for more than four hours in the absence of sexual arousal. The ischemic or low-flow subtype is encountered most frequently and represents an acute urological condition requiring prompt intervention. Sustained impairment of blood outflow results in tissue hypoxia and metabolic acidosis within the corpora cavernosa, which may subsequently cause smooth muscle injury, cavernosal fibrosis, and a substantial likelihood of irreversible erectile dysfunction.

Ischemic priapism constitutes a clinically serious condition that frequently necessitates progression from initial conservative management to more aggressive, invasive therapeutic strategies. Data from clinical studies suggest that extended periods of cavernosal ischemia are strongly associated with an increased likelihood of irreversible erectile impairment and sustained reductions in health-related quality of life. Contemporary guidelines and outcome analyses consistently identify the 24 h duration as a pivotal prognostic milestone influencing long-term functional recovery [4]. Therefore, priapism should not be regarded solely as an acute incident but rather as an event with the potential to adversely affect long-term somatic and psychosocial functioning [5].

Psychotropic medications play a significant role in the etiology of priapism, particularly antidepressants and antipsychotics. Data from recent years indicate that, despite a reduction in the number of indications for trazodone use, it remains one of the most frequently identified pharmacological causes of drug-induced priapism [6].

Previous studies have focused predominantly on the urological aspects of priapism, including episode duration, treatment efficacy, and the incidence of permanent erectile dysfunction. Far less attention has been devoted to long-term psychological and psychosocial consequences, despite the fact that the episode itself is often described by patients as highly stressful, humiliating, or traumatic [7].

An increasing body of evidence suggests that acute medical events involving the genital organs may result in persistent psychological impairments. Following episodes of priapism, patients report depressive and anxiety symptoms, reduced self-esteem, body image disturbances, avoidance of sexual activity, and difficulties with interpersonal relationships. Of particular importance are the fear of symptom recurrence and sustained anxiety related to pharmacotherapy [8].

In the case of trazodone-induced priapism, these issues acquire additional clinical significance. Such events occur in patients undergoing psychiatric treatment, often for mood disorders, anxiety disorders, or insomnia. Abrupt discontinuation of treatment, loss of trust in pharmacotherapy, and the need for urological intervention may contribute to destabilization of mental state and secondary deterioration of quality of life.

Despite the growing number of case reports, the literature lacks studies that systematically evaluate the long-term psychiatric and psychosocial sequelae of trazodone-induced priapism. Available data are predominantly descriptive and do not include standardized assessments of psychiatric symptoms or social functioning [7].

An episode of priapism meets the criteria for a highly stressful event, as it is sudden, intimate, and carries a real risk of permanent sexual dysfunction. Evidence from psychiatry and sexual medicine suggests that such events may contribute to the persistence of depressive and anxiety symptoms, development of avoidance behaviors, and reduced self-esteem. In patients treated with trazodone, an additional risk factor is the destabilization of antidepressant therapy following the incident, which may further exacerbate psychological functioning and overall quality of life [8].

Based on the available clinical data and the identified gap in the literature, the following research hypothesis was formulated: an episode of trazodone-induced priapism is associated with long-term psychiatric and psychosocial disturbances, including depressive and anxiety symptoms, sexual dysfunction, and reduced quality of life. This study aimed to conduct a clinical observational analysis of these sequelae in patients with a history of trazodone-induced priapism.

## 2. Materials and Methods

**Figure 1:**
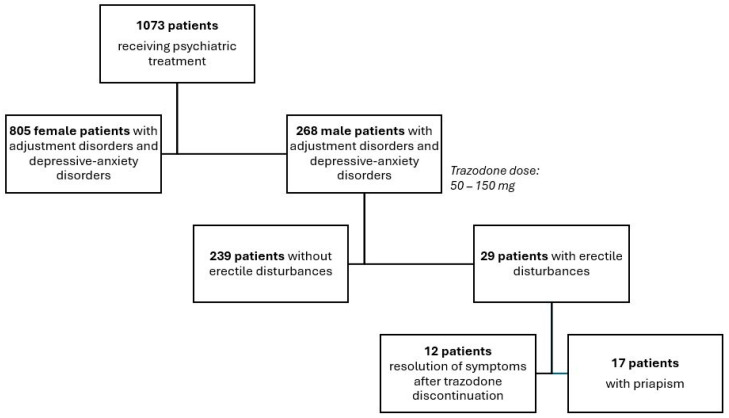
Study population receiving trazodone in clinical psychiatric practice.

The present study was observational and single-center in design, incorporating elements of prospective clinical follow-up. A total of 268 adult patients treated with trazodone, either hospitalized or managed in an outpatient setting, were included in the analysis. Within this cohort, 17 patients (6.3%) were hospitalized due to an episode of ischemic priapism, while 251 patients (93.7%) constituted a comparison group receiving trazodone without the occurrence of priapism (Figure 1).

The diagnosis of priapism was determined through an integrated assessment that included characteristic clinical features, persistence of penile rigidity beyond four hours, and findings from specialist urological evaluation, following internationally accepted standards for ischemic priapism management. The diagnosis of ischemic priapism was confirmed by a board-certified urologist based on clinical presentation, persistence of penile rigidity exceeding four hours, and specialist urological examination.

The inclusion criteria were as follows:

- Age ≥ 18 years;
- A confirmed episode of ischemic priapism;
- Trazodone use in the period preceding symptom onset;
- Feasibility of conducting a psychiatric assessment during hospitalization and throughout the follow-up period.

The exclusion criteria included:

- Priapism of a clearly established alternative etiology (e.g., hematological disorders);
- Coexisting acute psychotic disorders precluding reliable psychiatric assessment;
- Lack of patient consent to participate in follow-up observation.

These population selection criteria were consistent with approaches used in studies examining the sequelae of severe adverse effects of psychotropic medications [6,7,9].

All patients underwent psychiatric assessment during hospitalization and at predefined follow-up time points of 1, 3, and 6 months after the priapism episode. The follow-up schedule was designed to capture both early and persistent psychiatric and sexual sequelae, in accordance with recommendations from studies investigating the consequences of severe somatic and sexual adverse events [6].

The primary psychiatric outcomes included persistence or exacerbation of depressive and anxiety symptoms, while the primary sexual outcome was deterioration of erectile functioning following the priapism episode.

Clinical data related to the course of priapism were recorded in parallel, including episode duration, number of urological interventions, and level of treatment complexity. Alcohol or psychoactive substance exposure was assessed during structured clinical interviews and verified using available medical records. For analytical purposes, this variable was coded as present or absent.

Reported psychoactive substances included cannabis, benzodiazepines, and stimulant substances such as amphetamine derivatives.

Treatment complexity referred to the escalation and number of urological interventions required during priapism management, including corporal aspiration, intracavernosal sympathomimetic administration, and surgical shunting procedures The mean duration of the priapism episode was approximately 26 h (median 24 h), which falls within the time frame described in the literature as critical for the risk of permanent sexual and psychosexual sequelae [4,10].

Mental status and sexual functioning were assessed through structured clinical interviews conducted by trained clinicians, guided by widely validated psychometric frameworks. Depressive symptom severity was evaluated with reference to the Beck Depression Inventory–II (BDI-II) and the Hamilton Depression Rating Scale (HAM-D), while anxiety symptoms were assessed using the Generalized Anxiety Disorder-7 (GAD-7) framework. Erectile function was evaluated within the clinical interview using the International Index of Erectile Function-5 (IIEF-5) as a standardized reference. These instruments served as clinical reference frameworks guiding the structured assessment rather than formal questionnaire administration in all participants.

This approach, integrating patient-reported symptomatology elicited during clinician-led interviews with established psychometric constructs, allowed for a multidimensional evaluation of psychiatric and sexual sequelae. Such methodology is consistent with contemporary recommendations for research on psychosexual outcomes following acute urological events and drug-induced adverse reactions, particularly in settings where symptom interpretation and clinical context are essential.

Information regarding alcohol or recreational drug use at the time of the priapism episode was obtained from medical records and admission notes, based on patient self-report documented during clinical evaluation in the emergency department. Substance use was recorded as a binary variable (presence or absence of alcohol or drug use) when such information was available.

Statistical analyses were performed using GraphPad Prism 11.0 (La Jolla, CA, USA). Continuous variables are presented as mean ± standard error of the mean (SEM) or median with interquartile range (IQR), depending on the distribution of the data. Categorical variables are presented as counts and percentages.

Due to the imbalance in group sizes (17 vs. 251 participants), non-parametric statistical approaches were selected to minimize assumptions regarding distribution and variance. The control cohort was used primarily as a descriptive comparison rather than a strictly matched control population. Associations between continuous variables were explored using Spearman rank correlation coefficients. Comparisons between groups were conducted using the Mann–Whitney U test for continuous variables and Fisher’s exact test for categorical variables when appropriate.

To further explore potential factors associated with treatment intensity, an exploratory multivariable linear regression analysis was performed with the number of interventions required for priapism management as the dependent variable. Patient age, episode duration, and trazodone dose were included as explanatory variables.

A formal a priori sample size calculation was not feasible due to the unpredictable incidence of trazodone-associated priapism; therefore, all available cases within the study period were included.

All analyses were considered exploratory, and *p*-values < 0.05 were interpreted as indicative of potential statistical associations.

## 3. Results

The analysis included patients hospitalized due to ischemic priapism occurring during trazodone treatment, as well as a comparison group of patients receiving trazodone without a priapism episode. Patients with priapism were significantly older than those without priapism (44.1 ± 5.1 vs. 39.0 ± 4.4 years), as confirmed by statistical analysis (*p* < 0.0001). The distribution of trazodone doses (75, 100, and 150 mg) did not differ significantly between the groups; in the priapism group, doses of 75 mg and 150 mg predominated, while no cases of 100 mg dosing were observed. This finding is consistent with pharmacovigilance data, indicating that the risk of drug-induced priapism does not demonstrate a clear dose–response relationship and that individual susceptibility factors and duration of exposure may play a more critical role.

Patients with priapism were significantly older than those in the control group (44.1 ± 5.1 vs. 39.0 ± 4.4 years; *p* < 0.0001) as shown in Table 1.

The distribution of trazodone doses (75/100/150 mg) did not differ significantly between the groups, indicating that, within the analyzed cohort, medication dose itself did not constitute a differentiating factor for the risk of a priapism episode.

In the group of patients with priapism, substantial heterogeneity in the clinical course was observed, reflected by variability in the number of urological interventions and the degree of treatment complexity. The mean duration of the episode was 26.5 ± 16 h (median 24 h), representing a clinically significant value and consistent with literature data indicating that exceeding the 24 h threshold is associated with an increased risk of permanent sexual sequelae.

Spearman’s rank correlation analysis revealed predominantly weak-to-moderate associations among the analyzed clinical variables, with no single dominant relationship determining the clinical course.

**Figure 2:**
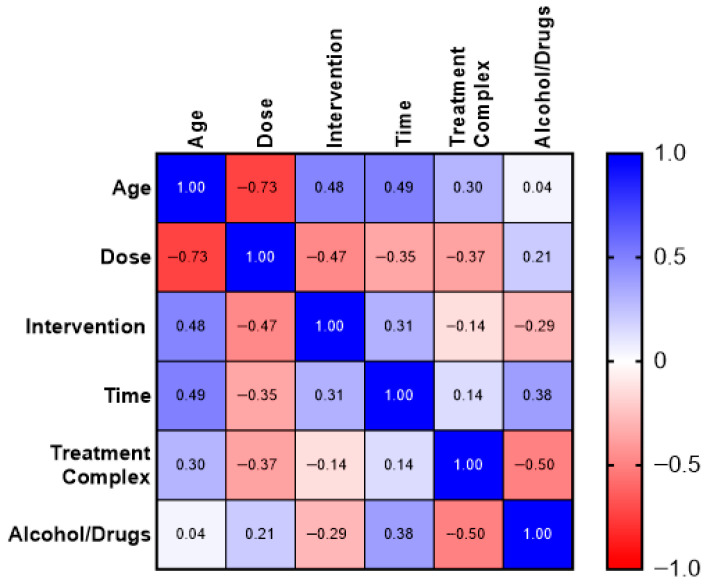
Correlation analysis of selected clinical variables in the priapism group.

The following findings were identified (Figure 2):

- A strong negative correlation between age and trazodone dose (ρ = −0.73), indicating that older patients more frequently received lower medication doses;
- A moderate positive correlation between age and the number of interventions (ρ = 0.48), as well as between age and episode duration (ρ = 0.49), suggesting a more severe and prolonged clinical course in older patients;
- A weak to moderate positive correlation between age and treatment complexity (ρ = 0.30);
- Moderate negative correlations between trazodone dose and the number of interventions (ρ = −0.47), episode duration (ρ = −0.35), and treatment complexity (ρ = −0.37), although these associations were descriptive in nature;
- A moderate positive correlation between the number of interventions and episode duration (ρ = 0.31), consistent with the escalatory nature of priapism management.

Treatment intensity was defined as the number of urological interventions and the escalation of treatment procedures required during priapism management.

Multivariable analysis demonstrated that a higher number of interventions was significantly associated with longer episode duration and older patient age, whereas trazodone dose was not an independent predictor of treatment intensity.

In exploratory analysis, the presence of alcohol or psychoactive substances was:

- Negatively correlated with the number of interventions (ρ = −0.29) and treatment complexity (ρ = −0.50);
- Positively correlated with episode duration (ρ = 0.38).

Trazodone dose showed no significant association with the presence of alcohol or psychoactive substances.

The observed negative correlations between trazodone dose and both episode duration and number of interventions may reflect covariation with patient age as well as clinical practice favoring more cautious dosing in older individuals.

Psychiatric assessments conducted during hospitalization and at follow-up time points of 1, 3, and 6 months demonstrated persistence of depressive and anxiety symptoms following the priapism episode. The observed symptom trajectory indicated that, in a subset of patients, these symptoms were not confined to the acute phase but persisted over the longer term.

Key Findings of the Study:

- Patients who experienced priapism were significantly older than those who received trazodone without priapism, whereas trazodone dose distribution (75/100/150 mg) did not differ significantly between groups.
- The clinical course of priapism was characterized by substantial heterogeneity, including variability in the number of urological interventions and treatment complexity; mean episode duration was approximately 26 h (median 24 h).
- Spearman’s rank correlation analysis revealed predominantly weak-to-moderate associations among analyzed clinical variables, with no single dominant relationship determining the clinical course.
- Patient age was positively correlated with episode duration, number of interventions, and treatment complexity, indicating a more severe and prolonged course in older patients.
- Trazodone dose showed moderate negative correlations with number of interventions, episode duration, and treatment complexity. However, these associations were descriptive in nature and did not support an independent effect of dose on treatment intensity.
- Number of interventions was positively correlated with episode duration, consistent with the escalatory nature of ischemic priapism management.
- Presence of alcohol or psychoactive substances was negatively correlated with number of interventions and treatment complexity, while positively correlated with episode duration.
- Multivariable analysis demonstrated that treatment intensity was significantly associated with longer episode duration and older age, whereas trazodone dose was not an independent predictor.
- Associations including alcohol or psychoactive substances and treatment intensity most likely reflect differences in clinical course or management strategies rather than direct causal effects, potentially arising from variable construction and treatment decision pathways.
- Analysis of alcohol and psychoactive substance use was exploratory and indicated associations with age and episode duration, without significant influence of trazodone dose.

## 4. Discussion

In the present analysis, an episode of trazodone-associated priapism was not merely an acute urological emergency but was associated with consequences extending beyond the hospitalization period. From a clinical perspective, older patient age and longer episode duration were correlated with greater treatment intensity, reflected by the number of interventions and treatment complexity, whereas trazodone dose did not emerge as an independent predictor of a more severe clinical course in multivariable analysis.

From the perspective of the present dataset, several of the observed associations deserve particular attention. In this cohort, older patient age and longer episode duration were consistently related to a more intensive therapeutic course, reflected by a greater number of urological interventions and higher treatment complexity. At the same time, trazodone dose did not appear to influence episode severity in the multivariable model. These observations highlight that the clinical course of trazodone-associated priapism in this study was primarily determined by patient-related and episode-related factors rather than by the pharmacological exposure itself. Interpreting the results in this way allows the findings of the present analysis to be viewed not only as a description of individual cases but also as a structured characterization of the clinical course and subsequent psychiatric and sexual outcomes following this adverse drug reaction.

This finding is consistent with the established paradigm that prognosis in ischemic priapism is determined primarily by the duration of ischemia and delays in effective urological intervention, rather than by a single parameter of pharmacological exposure.

Concurrently, in the psychiatric domain, consistent with the predefined assumptions and assessments conducted at multiple follow-up time points, a significant exacerbation of depressive and anxiety symptoms, along with deterioration in sexual functioning, was observed following the incident. Clinically, this may be interpreted as a cumulative effect of: (1) an acute and sudden event threatening sexual function; (2) subsequent prognostic uncertainty regarding erectile recovery; (3) perceived loss of control and experiences of stigmatization; and (4) potential destabilization of antidepressant treatment following modification or discontinuation of therapy.

Pharmacovigilance data and clinical reviews consistently indicate that psychotropic medications—including trazodone—are among the most frequently reported causes of drug-induced priapism, with the mechanism primarily attributed to α1-adrenergic receptor antagonism leading to impaired detumescence. At the same time, the dominant body of literature focuses on diagnosis, acute management algorithms, and somatic complications (e.g., erectile dysfunction or the need for surgical intervention), whereas the psychiatric dimension is most frequently marginalized or addressed only secondarily.

Recent work in the field of sexual medicine, particularly studies appearing in contemporary andrological, urological, and sexology-focused journals, increasingly indicates that the impact of priapism is not limited to urological outcomes alone. Instead, it also involves sustained effects on overall quality of life, relational functioning, and mental health. The results of the present study are consistent with this evolving view, indicating that an episode of priapism may represent a significant life stressor, followed by persistent depressive and anxiety symptoms, with sexual dysfunction becoming strongly influenced by psychosocial factors.

Trazodone-induced priapism typically occurs in patients with pre-existing psychiatric indications, such as insomnia, depressive disorders, or anxiety disorders. This implies reduced psychological reserve and increased vulnerability to symptom persistent symptoms following a stressful event. Moreover, an incident that is sudden, intimate, and potentially stigmatizing may trigger mechanisms of avoidance behaviors, shame, anticipatory anxiety, and diminished self-esteem, particularly when patients perceive a genuine risk of permanent erectile dysfunction.

An additional contributing factor is the discontinuation or substantial modification of antidepressant treatment following the incident. In clinical practice, this may create a feedback loop: (1) adverse event; (2) medication discontinuation; (3) worsening of primary psychiatric symptoms; and (4) secondary deterioration in psychosocial and sexual functioning. From a pharmacotherapy safety perspective, this is particularly relevant, as severe sexual adverse events carry a high potential to undermine long-term patient trust in treatment. In this context, the exploratory associations observed with alcohol or psychoactive substance use should be interpreted with caution. These relationships may reflect differences in healthcare-seeking behavior, clinical course, or therapeutic decision-making rather than direct causal effects. Similar descriptive patterns have been reported in pharmacovigilance analyses and observational studies, supporting the interpretation that such findings are influenced by patient and treatment characteristics rather than independent risk mechanisms [6].

These findings support an interdisciplinary approach, whereby standard urological care following priapism should be complemented by early psychiatric screening and, when indicated, targeted psychological or psychiatric interventions. In clinical practice, this may include:

- Brief assessment of depressive and anxiety symptoms during hospitalization and follow-up visits;
- Psychoeducation regarding recurrence risk, sexual consequences, and the monitoring strategies;
- Support in decision-making related to continuation or modification of antidepressant pharmacotherapy;
- Consideration of interventions targeting anticipatory anxiety and avoidance behaviors (e.g., elements of cognitive-behavioral therapy);
- Inclusion of the partner perspective, when applicable.

The literature on priapism, including classical clinical reviews, emphasizes that even optimally conducted urological interventions do not eliminate the risk of long-term sequelae. Present data extend this observation by incorporating a psychiatric dimension, indicating that successful acute management does not necessarily equate to a return to baseline psychological and sexual functioning [7,8,11,12].

Key limitations of this study include its observational design and the relatively small size of the priapism subgroup, which may reduce statistical power to detect more subtle associations (e.g., dose–risk relationships). This reflects the rarity of this adverse drug reaction in clinical practice. Consequently, the study should be interpreted primarily as an exploratory observational analysis rather than a definitive comparative cohort study. The larger control group was included to provide contextual clinical characteristics of trazodone-treated patients without priapism, rather than to establish a strictly matched case–control comparison. Additionally, the incompleteness of clinical data in some cases, reflecting real-world conditions of acute hospitalization and follow-up, may have limited the availability of certain psychiatric and clinical assessments across all observation points. Potential confounding factors should also be considered, including concomitant medication use, exposure to psychoactive substances, baseline severity of depressive and anxiety symptoms, and variability in the time to initiation of urological treatment. Psychiatric and sexual outcomes were assessed through structured clinician-led interviews informed by established psychometric frameworks (BDI-II, HAM-D, GAD-7 and IIEF-5) rather than through formal standardized questionnaire administration in all participants.

A key strength of the present study lies in its focus on the systematic assessment of psychological and sexual functioning at predefined follow-up time points and the integration of these findings with clinical data related to the course of priapism, including episode duration and number of interventions.

Both pharmacovigilance reports and clinical reviews underscore the need for improved understanding of risk factors and the sequelae associated with drug-induced priapism. From the perspective of the study hypothesis, future prospective research is particularly warranted to:

- Include larger cohorts of patients with trazodone-induced priapism;
- Incorporate baseline psychiatric assessments, when feasible, and standardized follow-up measures (e.g., BDI-II/HAM-D/GAD-7, along with quality-of-life and post-traumatic stress measures);
- Enable modeling of persistent outcome risk as a function of ischemia duration, type of intervention, and patient profile;
- Evaluate the impact of antidepressant discontinuation or modification on the trajectory of psychiatric symptoms.

## 5. Conclusions

Trazodone-associated priapism should be regarded not only as an acute urological emergency but also an event with potentially significant psychiatric and psychosocial consequences extending beyond the acute phase. Evidence from this study supports the view that episode severity, particularly ischemia duration, and patient age influence clinical course and treatment intensity, whereas trazodone dose alone does not appear to be an independent determinant of outcome. Importantly, priapism may followed by persistent depressive and anxiety symptoms, deterioration in sexual functioning, and destabilization of antidepressant therapy, underscoring the need for comprehensive post-episode care. An interdisciplinary approach integrating urological management with early psychiatric assessment, patient education, and monitoring of mental health and sexual functioning may help mitigate long-term adverse sequelae and improve overall quality of life.

## Figures and Tables

**Table 1:** Basic characteristics of the cohort. Data shown as mean ± SEM and median (full range).

Parameter	Priapism (n = 17)	Without Priapism (n = 239)	*p* Value
Age (Years)	44.1 ± 5.12 44.0 (34.0–55.0)	39.0 ± 4.44 39.0 (31.0–46.0)	<0.0001
Average Daily Dose of Trazodone	124 ± 8.96	120 ± 2.12	0.6453
Dose of Trazodone (75/100/150) mg daily	6/0/11	71/46/134	0.1522
Number of Interventions	5/9/3	N/A	
Treatment Complexity	5/10/2	N/A	
Alcohol/Drugs (Y/N)	5/12	N/A	
Time [h]	24 (19–33)	N/A	
Drug Abuse (Cocaine-Positive test)	2/15	N/A	
Under the Influence of Alcohol (Yes)	5/12	N/A	

## Data Availability

Data is available through the Corresponding Author upon reasonable request.

## Abbreviations

The following abbreviations are used in this manuscript:

SARI	Serotonin receptor antagonists and serotonin reuptake inhibitors
BDI-II	Beck Depression Inventory–II
HAM-D	Hamilton Depression Rating Scale
GAD-7	Generalized Anxiety Disorder-7
IIEF-5	International Index of Erectile Function-5
